# Tracking the evolution of the SARS-CoV-2 Delta variant of concern: analysis of genetic diversity and selection across the whole viral genome

**DOI:** 10.3389/fmicb.2023.1222301

**Published:** 2023-08-08

**Authors:** Katherine Li, Stephanie Melnychuk, Paul Sandstrom, Hezhao Ji

**Affiliations:** ^1^National Microbiology Laboratory at JC Wilt Infectious Diseases Research Centre, Public Health Agency of Canada, Winnipeg, MB, Canada; ^2^Department of Medical Microbiology and Infectious Diseases, Max Rady College of Medicine, University of Manitoba, Winnipeg, MB, Canada

**Keywords:** SARS-CoV-2, evolution, genetic diversity, selection, whole genome

## Abstract

**Background:**

Since 2019, severe acute respiratory syndrome coronavirus 2 (SARS-CoV-2) has diversified extensively, producing five highly virulent lineages designated as variants of concern (VOCs). The Delta VOC emerged in India with increased transmission, immune evasion, and mortality, causing a massive global case surge in 2021. This study aims to understand how the Delta VOC evolved by characterizing mutation patterns in the viral population before and after its emergence. Furthermore, we aim to identify the influence of positive and negative selection on VOC evolution and understand the prevalence of different mutation types in the viral genome.

**Methods:**

Three groups of whole viral genomes were retrieved from GISAID, sourced from India, with collection periods as follows: Group A—during the initial appearance of SARS-CoV-2; Group B—just before the emergence of the Delta variant; Group C—after the establishment of the Delta variant in India. Mutations in >1% of each group were identified with BioEdit to reveal differences in mutation quantity and type. Sites under positive or negative selection were identified with FUBAR. The results were compared to determine how mutations correspond with selective pressures and how viral mutation profiles changed to reflect genetic diversity before and after VOC emergence.

**Results:**

The number of mutations increased progressively in Groups A–C, with Group C reporting a 2.2- and 1.9-fold increase from Groups A and B, respectively. Among all the observed mutations, Group C had the highest percentage of deletions (22.7%; vs. 4.2% and 2.6% in Groups A and B, respectively), and most mutations altered the final amino acid code, such as non-synonymous substitutions and deletions. Conversely, Group B had the most synonymous substitutions that are effectively silent. The number of sites experiencing positive selection increased in Groups A–C, but Group B had 2.4- and 2.6 times more sites under negative selection compared to Groups A and C, respectively.

**Conclusion:**

Our findings demonstrated that viral genetic diversity continuously increased during and after the emergence of the Delta VOC. Despite this, Group B reports heightened negative selection, which potentially preserves important gene regions during evolution. Group C contains an unprecedented quantity of mutations and positively selected sites, providing strong evidence of active viral adaptation in the population.

## 1. Introduction

Severe acute respiratory syndrome coronavirus 2 (SARS-CoV-2), a betacoronavirus infamous for causing the coronavirus disease 2019 (COVID-19), emerged in late 2019 and rapidly escalated into a global pandemic. The SARS-CoV-2 genome spans approximately 30 kb in length and encodes 16 non-structural, four structural, and nine accessory proteins (Wu et al., 2020; Bai et al., 2022). The structural proteins include the nucleocapsid (N), membrane (M), envelope (E), and spike (S) (Khailany et al., 2020). Glycosylated S proteins cover the virion surface and contain the receptor-binding domain (RBD), which mediates host-binding interactions (Huang et al., 2020; Letko et al., 2020). The E protein contributes to the formation of the viral envelope, while the M protein plays an essential role in virion assembly (Bai et al., 2022). The N protein binds the viral genome and has multiple roles in RNA synthesis and translation, viral replication, and cell cycle regulation (Bai et al., 2022). The ORF1ab gene encodes 16 non-structural proteins that are essential for viral RNA replication and transcription (Bai et al., 2022). The accessory proteins encoded by other open reading frames (ORFs) encompass a diverse range of functions, including viral release, inhibition of host cellular functions and immune response, formation of ion channels, and interactions with other viral proteins (Li et al., 2020; Toft-Bertelsen et al., 2021; Zinzula, 2021; Bai et al., 2022).

SARS-CoV-2, such as other RNA viruses, exhibits a high mutation rate primarily attributed to its error-prone RNA-dependent RNA polymerase (Duarte et al., 2022). When coupled with high rates of viral reproduction, these mutations may occur throughout the viral genome, allowing viral quasi-species to arise and persist within the infected host (Karamitros et al., 2020). Additionally, exposure to an enormous pool of susceptible hosts favors the rapid evolution of SARS-CoV-2, producing numerous viral variants that circulate within human populations (Duarte et al., 2022). This has been well illustrated by the many SARS-CoV-2 variants of concern (VOCs) designated by the World Health Organization (WHO), which possess mutations that confer high-risk traits including increased transmissibility, increased virulence, and reduced susceptibility to vaccines and therapeutics (World Health Organization, 2021). The designated VOCs included the Alpha, Beta, Gamma, Delta, and Omicron lineages (World Health Organization, 2021). In particular, the Delta VOC has been known for its increased virulence, pathogenicity, and severity of COVID-19 disease (Liu and Rocklöv, 2021; Zhang et al., 2022).

The first SARS-CoV-2 infection case in India was reported in Kerala on 30 January 2020 (Andrews et al., 2020). On 11 March 2020, the WHO declared COVID-19 a global pandemic, prompting the Indian government to impose a nationwide lockdown on 25 March 2020 (Siddiqui et al., 2020). A subsequent wave of transmission hit India during the Spring of 2021, driven by the emergence of a new variant, B.1.617.2, in Maharashtra (Rambaut et al., 2020). B.1.617.2 was later designated as a VOC in May 2021, following a significant surge in cases both regionally and globally, and was subsequently renamed the Delta variant (World Health Organization, 2021). Retrospective investigations revealed that the Delta VOC first emerged in India in mid-September 2020, although its transmission did not escalate until March 2021 (McCrone et al., 2022). The surge in Delta cases was accompanied by an alarming increase in local mortality and hospitalization rates due to its heightened transmissibility and immune evasion (Zhan et al., 2022). These advantageous features allowed Delta VOC to overtake the Alpha VOC as the dominant global lineage, fueling new outbreaks and resurgences worldwide despite the advancement of vaccine uptake (Liu and Rocklöv, 2021; Zhang et al., 2022).

In this study, we aim to characterize the genetic diversity and evolutionary trends of the Delta VOC in India. To achieve this, we conducted a multilayered analysis of the SARS-CoV-2 genome using archived whole-genome SARS-CoV-2 sequences from a public database. By collecting database-derived sequences at three distinct time periods surrounding the emergence of Delta VOC in India, we aim to identify specific mutation patterns that characterize the state of the viral population immediately before VOC emergence and reflect the succession of Delta VOC as the dominant SARS-CoV-2 lineage in early 2021. Furthermore, we seek to identify genomic regions of SARS-CoV-2 that underwent positive or negative selection to further improve our understanding of the SARS-CoV-2 evolutionary progression.

## 2. Materials and methods

### 2.1. Establishment of study groups and data collection

**Figure 1:**
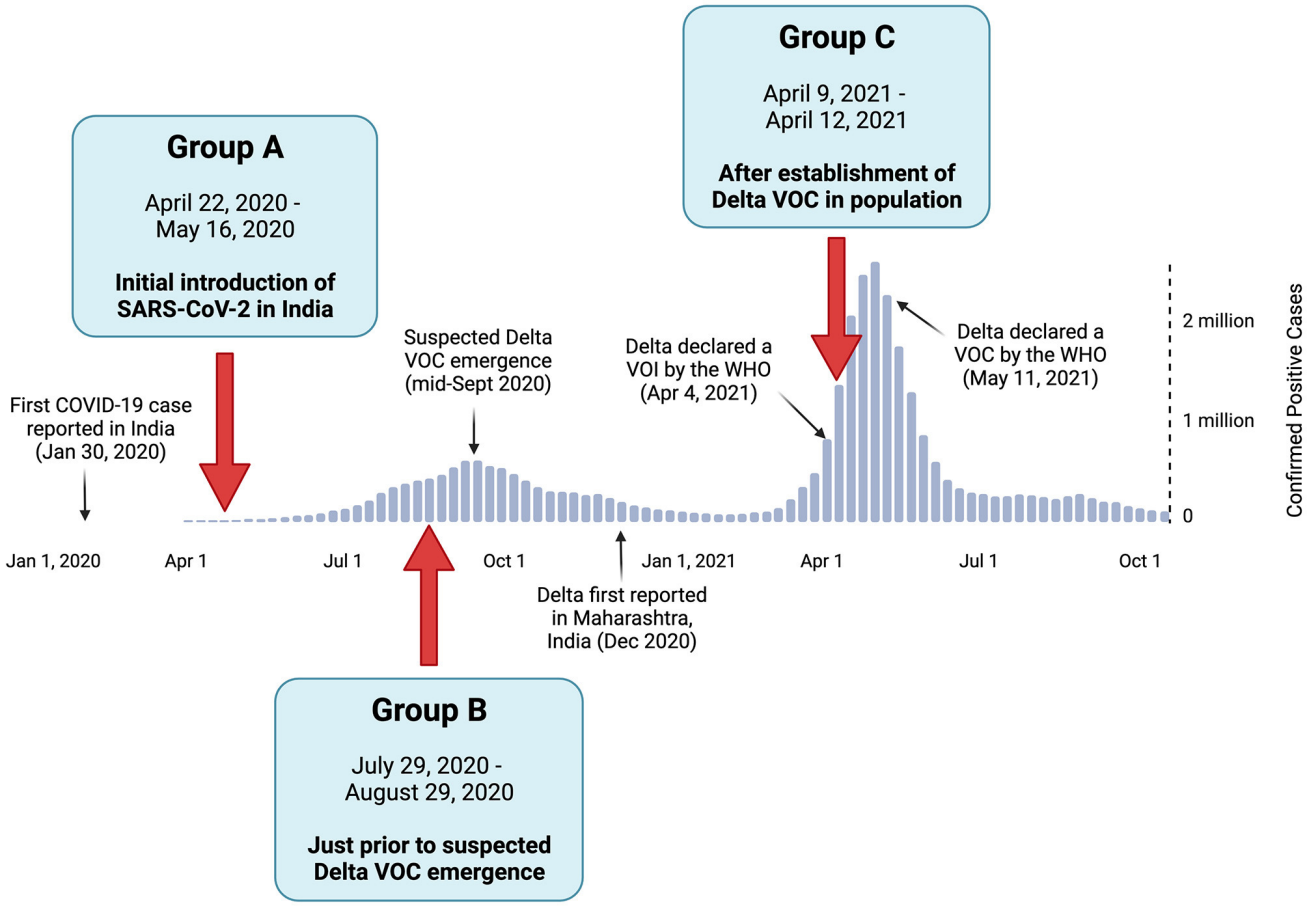
Positive COVID-19 case summary in India, with the relative timeline of events pertaining to the Delta variant of concern (VOC). The positive case numbers were based on the World Health Organization reports (World Health Organization, 2020). The collection dates and rationale of study Groups A, B, and C are denoted by red arrows.

Full-length SARS-CoV-2 genomes derived from clinical specimens in India were sourced from the GISAID database for three study groups (A, B, and C) based on their initial sampling dates (Khare et al., 2021). The study groups and their corresponding time periods of interest included (1) Group A, which represents the earlier stage of the COVID-19 pandemic in India, when all reported cases were caused by the initial SARS-CoV-2 Wuhan strain and its early descendants; (2) Group B, which reflects the pre-Delta period, which was immediately before the suspected emergence of Delta VOC; and (3) Group C, which corresponds to the Delta-dominant period, when the Delta VOC was well established in the population (Figure 1) (India Today Web Desk, 2020; World Health Organization, 2020; Choudhary et al., 2021). The duration of the study periods was adjusted to ensure the sample size of each group was both acceptable and comparable to the other groups, as the number of positive COVID-19 cases per day (and thus the available genomes) varied greatly among the time periods of the three groups.

**Figure 2:**
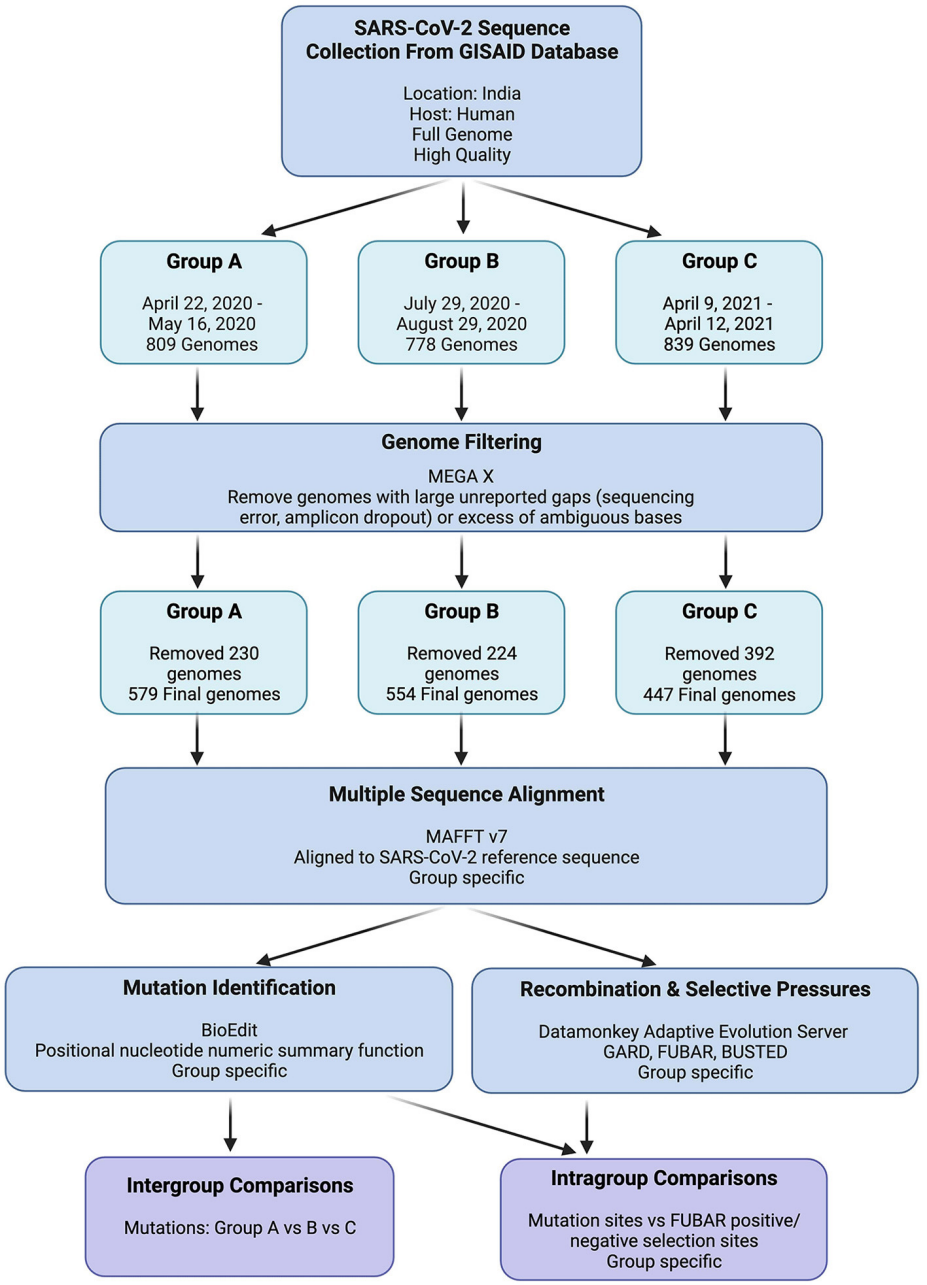
GISAID SARS-CoV-2 genome data retrieval and processing workflow. “Group Specific” indicates that individual analyses were performed on Groups A, B, and C independently.

To ensure the reliability and completeness of the SARS-CoV-2 genome data used in our analysis, the GISAID filters for high-quality and complete genome sequences were applied while retrieving viral genomes for each group. The retrieved viral genomes were then viewed in Molecular Evolutionary Genetics Analysis (MEGA X), which revealed multiple genomes with indications of poor sequence quality (Kumar et al., 2018). These indicators included large unreported gaps (suggestive of amplicon dropout) or an excessive number of ambiguous bases, potentially arising from sequencing errors. Considering the potential interference of unreliable sequences in our mutation and selection investigations, these genomes were excluded from further analyses if the indicators at any given locus exceeded 1% of the total group's sequences. The detailed data retrieval and processing workflow is depicted in Figure 2.

### 2.2. Multiple sequence alignment

For each study group, all viral genome sequences were aligned to the original Wuhan-Hu-1 SARS-CoV-2 reference sequence (GISAID Accession EPI_ISL_402124) using the MAFFT v7 online server option for SARS-CoV-2 (https://mafft.cbrc.jp/alignment/server/add_sarscov2.html?mar15) (Katoh et al., 2019). The derived multiple sequence alignment (MSA) files were visually inspected in MEGA X (Kumar et al., 2018).

### 2.3. Mutation identification

Each group's full genome MSA was used to generate a positional nucleotide numeric summary file in BioEdit (Hall, 1999). This output file was then split into the individual genes of SARS-CoV-2 using an in-house Python program (https://github.com/ connor-lowey/SARS-CoV-2_Delta_Helper_Scripts). To determine the prevalence of the mutations, the frequencies of all identified genetic variations were calculated and categorized using thresholds of < 1%, 1–10%, and >10% for each respective group. The chosen thresholds stopped at >10% to accommodate heterogeneous viral populations and to avoid excluding an excess of data by setting an unattainable threshold. Mutations across the whole viral genome were characterized for all groups by recording mutations present at each frequency threshold in each individual gene relative to the SARS-CoV-2 reference genome. Potential codon changes at the amino acid level were recorded as resulting in a synonymous or non-synonymous amino acid substitution, an insertion, or a deletion. All mutations within a given gene at both the nucleotide and amino acid levels were compared among groups to determine which mutations were shared and which were unique to a particular group. The intergroup differences in mutation prevalence were further assessed for statistical significance using an in-house Python script (https://github.com/ connor-lowey/SARS-CoV-2_Delta_Helper_Scripts). A chi-squared test of independence was used to compare groups by individual gene, and a Bonferroni correction was applied. A *p* < 0.05 was considered statistically significant.

### 2.4. Recombination and selection

To analyze recombination events and evidence of natural selection, we processed each group's whole-genome MSA file by splitting it into individual gene ORFs using MEGA X and then tested them for recombination and evidence of selection on the Datamonkey webserver (Kumar et al., 2018; Weaver et al., 2018). The recombination events were first assessed using the Genetic Algorithm for Recombination Detection (GARD) with site-to-site rate variation set to *General Discrete* and rate classes set to three (Kosakovsky Pond et al., 2006). All other parameters were left as default settings. Before conducting selection analyses, all stop codons (terminal and premature) were removed from the MSA files with MEGA X. This step was necessary as the Datamonkey selection tools reject the presence of any stop codons (Kumar et al., 2018). Gene-specific MSA files, omitting stop codons, were used to detect site-specific positive/negative selection using the Fast, Unconstrained Bayesian AppRoximation for Inferring Selection (FUBAR) tool with default parameter settings (Murrell et al., 2013). All amino acid sites under positive or negative selection were compared against the list of mutations in the corresponding group to determine which sites under selection coincided with a mutation present at a frequency of >1% in the group. The intergroup differences in the quantities of positive and negative selection sites were assessed for statistical significance using an in-house Python script, as described in the section above.

## 3. Results

### 3.1. Establishment of study groups

The collection dates for each of the three study groups were selected based on the timeline of SARS-CoV-2 and the emergence of Delta VOC in India. As shown in Figure 1, Group A contained sequences collected from 22 April to 16 May 2020 (*n* = 579) during the initial introduction of the SARS-CoV-2 Wuhan strain to India. Group B was sampled from 29 July to 29 August 2020 (*n* = 554), representing the period just before the suspected emergence of the Delta VOC. Group C contained sequences from 9 April to 12 April 2021 (*n* = 447) during the surge in positive cases predominantly attributed to the Delta VOC. The most prevalent viral lineage found in Group A was B.1, which was the dominant global lineage during the start of the pandemic (Rambaut et al., 2020). In contrast, the most prevalent lineages were B.1.1.306 in Group B (a descendent lineage that did not achieve any notable WHO designations) and B.1.617.2 in Group C (the Delta VOC, as designated by the WHO). This highlights the diversification away from the original strain over time (Rambaut et al., 2020; World Health Organization, 2021).

### 3.2. Mutation analysis

**Figure 3:**
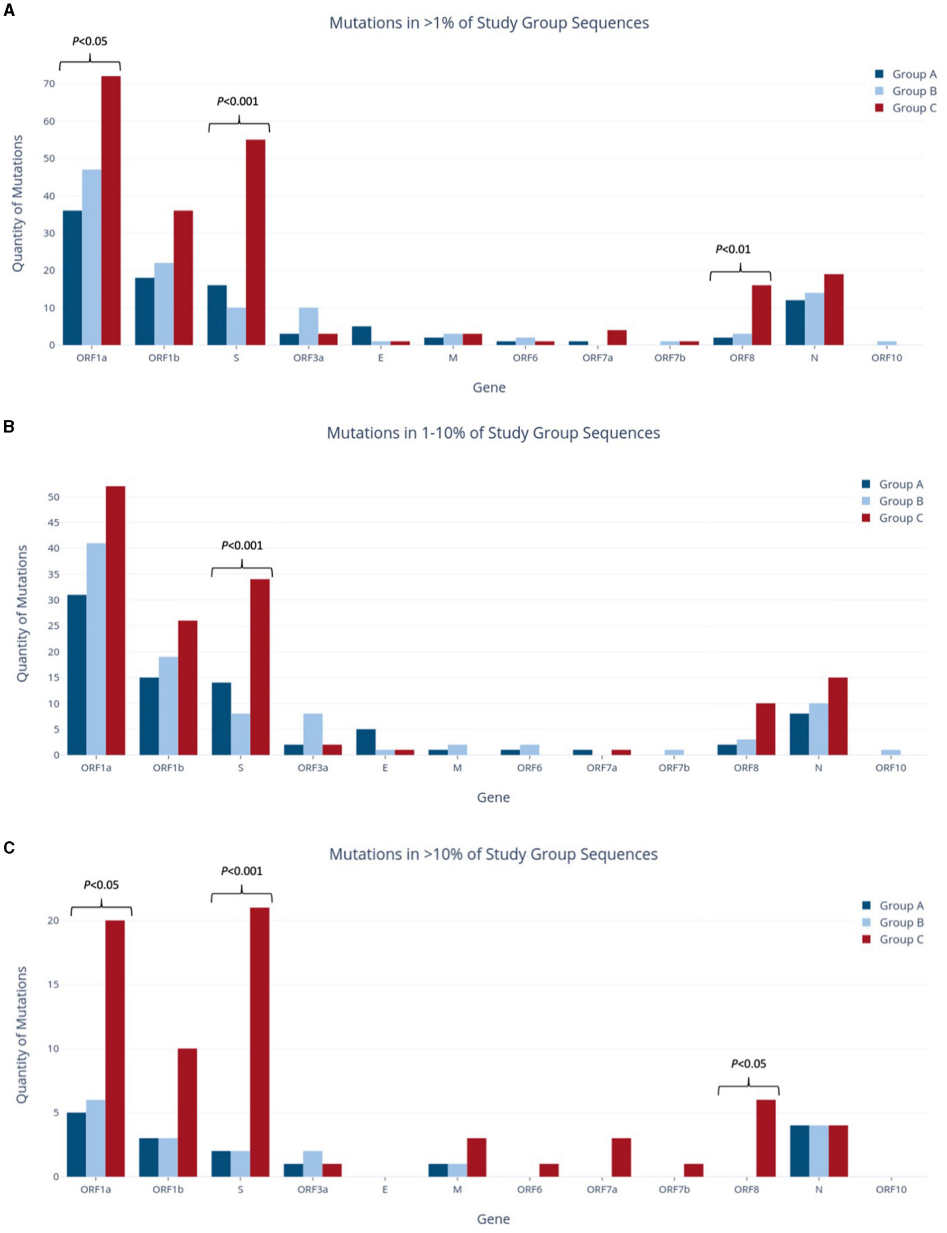
Quantities of nucleotide-level mutations identified in study group sequences at relative frequencies of >1% **(A)**, 1–10% **(B)**, and >10% **(C)**.

To assess the temporal progression of mutation frequency and type across Groups A, B, and C, we first identified all mutations that are present at frequencies >1% in each of the study groups. Further mutation profiling revealed varying proportions of non-synonymous and synonymous codon changes among the groups, with Group C showing more distinctive results while Groups A and B were more alike. In all study groups, the ORF1a gene had the largest number of mutations out of any gene, as it makes up over 40% of the viral genome (Figure 3A) (Khare et al., 2021). Following closely behind in both Groups A and C were ORF1b and the S gene, as well as the N gene.

**Figure 4:**
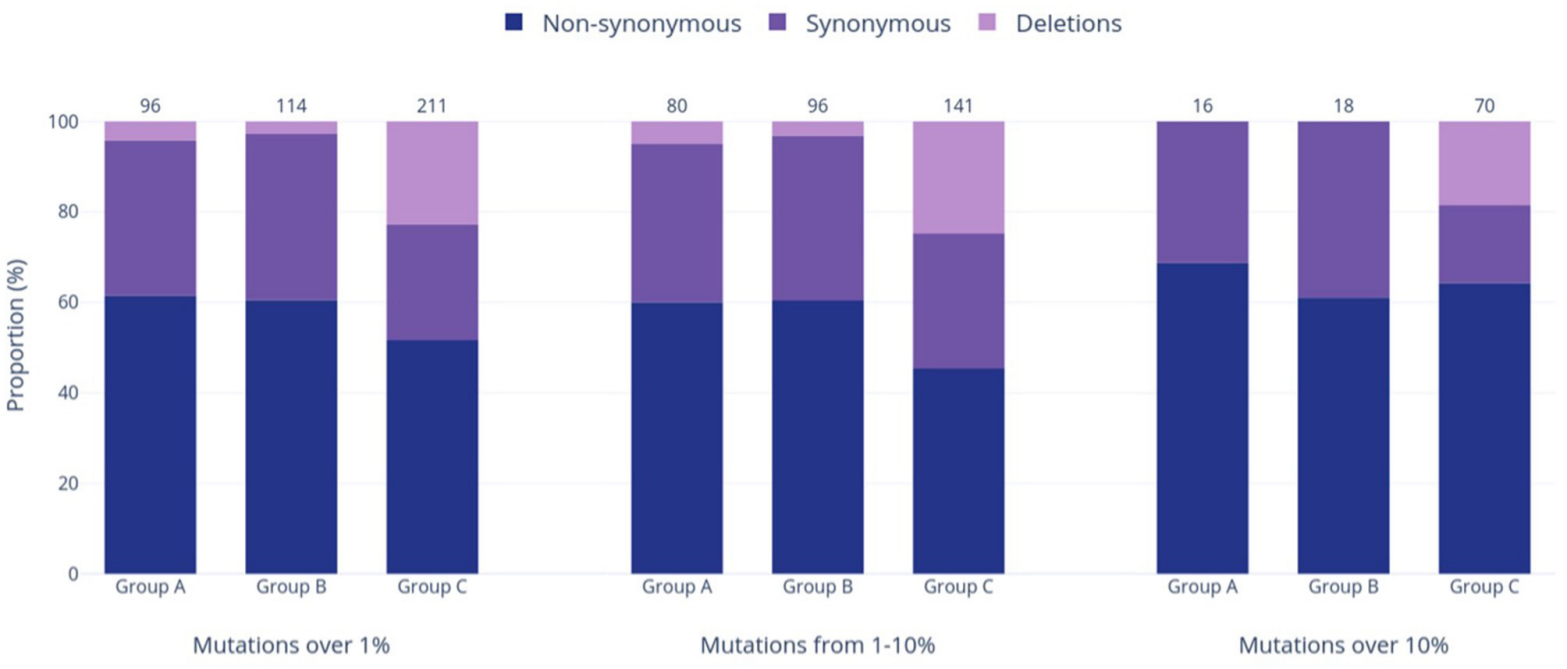
Prevalence of varied genetic mutation types in the three study groups at relative frequencies of >1%, 1–10%, and >10%. The bars in the graph represent the occurrence of each mutation type, while the numbers above the bars show the total counts of mutations within each respective group and frequency range.

Group A was found to have the lowest number of nucleotide mutations (96), including the lowest quantity of both non-synonymous and synonymous substitutions out of all groups (Figure 4). However, when the relative percentage of each mutation type was calculated for the study groups, Group A had the largest proportion of non-synonymous mutations out of all the study groups. Specifically, this mutation type made up 61.5% of mutations in Group A and 60.5% and 51.7% of mutations in Groups B and C, respectively. Forty-eight (60.0%) of all mutations present at the 1–10% sequence threshold and 11 (68.8%) of all mutations present at the >10% sequence threshold encoded a non-synonymous substitution (Figure 4). Of these, the greatest number of non-synonymous substitutions in Group A occurred in ORF1a, with 17 at the 1–10% threshold range and 3 at the >10% threshold (Supplementary Table 1A). Notably, Group A had no insertions and only four deletions, all occurring in the E gene at the 1–10% threshold (Supplementary Table 1A).

Group B exhibited an intermediate quantity of mutations, with a higher total count than Group A but fewer mutations than Group C across all frequency ranges (Figures 3A–C). Out of the 114 mutations in Group B, 96 (84%) fell within the 1–10% frequency range, while only 18 mutations (16%) occurred in >10% of the group's sequences. This proportion of mutations reaching the >10% threshold was the lowest compared to the other groups (17% in Group A, 33% in Group C), although Group B had noticeably more mutations overall than Group A (114 vs. 96 mutations >1%, respectively). Most mutations were present in ORF1a and ORF1b, followed next in quantity by the N gene rather than the S gene (as seen in Groups A and C). Group B had the highest relative proportion of synonymous mutations across all frequency ranges (37.8% overall, Figure 4), most of which were found in ORF1a (Supplementary Table 1B). Similar to Group A, Group B did not contain any insertions and had the fewest deletions among all groups.

Group C had the highest mutation count at all frequency ranges among the three study groups (Figures 3A–C), with 2.2 and 1.85 times more mutations than the Group A and B totals at the >1% level, respectively. These differences were statistically significant, with *p* < 0.001 for both pairwise comparisons of Groups A and B vs. Group C. Similarly, this group had significantly more mutations present at a frequency of >10%, with 4.4 and 3.9 times more than Groups A and B (*p* < 0.001), respectively. Interestingly, Group C also had the highest number of deletions present at all frequencies, and these deletions made up 22.7% of the group's total mutations shown in Figure 4. Consequently, the relative proportions of non-synonymous and synonymous substitutions were observed at lower amounts in Group C in comparison to the other groups; however, Group C had more mutations that cause a change in the amino acid coding (sum of non-synonymous substitutions, insertions, and deletions) than any other group at all frequencies (74% of all mutations present in >1% of sequences). Most of Group C's deletions were found in the ORF1a, ORF1b, S, and ORF8 genes (Supplementary Table 1C). Compared to Groups A and B, Group C contained significantly larger mutation counts in the S gene at all frequency levels (*p* < 001). At a frequency of >10%, the S gene in Group C had one more mutation than ORF1a (21 and 20, respectively), despite ORF1a being 3.5 times longer than the S gene (Khare et al., 2021). Similarly, Group C also reported a significant difference in the number of mutations in ORF1a and ORF8 when compared to other study groups, as shown in Figure 3A (*p* < 0.05 and *p* < 0.01, respectively).

All mutations occurring in >1% of their respective group were compared to determine common and unique mutations within Groups A–C. Across the entire genome, a total of 42 amino acid coding sites were affected by mutations shared by at least two of the three study groups (Table 1). These involved 49 different nucleotide-level mutations, accounting for instances where multiple mutations occurred within the same codon. Notably, ORF7a, ORF7b, and ORF10 were the only genes that contained no shared mutations between study groups. On the other hand, ORF1a alone had almost half (48%) of the shared mutations in the entire genome, with 20 shared amino acid sites resulting from 23 unique nucleotide-level mutations. ORF1b and N held the next-largest numbers of shared amino acid sites (seven and five, respectively), although these were considerably lower than the quantity of ORF1a. The most common combination for shared mutations was Group A and Group B, accounting for 25 of the 42 possible amino acid sites. Nine amino acid sites contained mutations shared by Groups A, B, and C together, while six amino acid sites had mutations shared by just Groups B and C. The least frequent pairing was Group A with Group C, which only occurred at two amino acid sites in the full genome.

**Table 1:** Quantities of mutated amino acid sites shared by multiple study groups.

**Gene**	**Mutated amino acid sites shared by ≥2 study groups**	**Unique mutations^a^ affecting shared amino acid sites**
ORF1a	20	23
ORF1b	7	8
S	4	5
ORF3a	2	2
E	1	1
M	1	1
ORF6	1	1
ORF7a	0	0
ORF7b	0	0
ORF8	1	1
N	5	7
ORF10	0	0
Full genome	42	49

^a^Mutations, reflecting a single mutation at the nucleotide level where multiple distinct mutations (substitutions and/or deletions) can occur within the same amino acid codon.

To quantify the unique mutations that were specific to each study group (not shared with another group), comparisons were made using genes, and the results are summarized in Table 2. Notably, unique mutations made up most of the mutations identified in Group C, at 91.5% (193 of the total 211 mutations present in >1% of Group C). The proportions of Group A and B were considerably lower than Group C, with unique mutations comprising 60.4% and 63.2% of all mutations in each group, respectively. Many of Group C's unique mutations were found in ORF1a, ORF1b, and the S gene. In ORF1a, Group C had 2.4- and 3.3 times more unique mutations than Groups B and A, respectively. Similarly, in ORF1b, Group C had 2.1- and 2.9 times more unique mutations than Groups B and A, respectively. The S gene of Group C exhibited a strikingly higher unique mutation count, with 4.3- and 7.4 times more mutations than those identified in Groups A and B, respectively.

**Table 2:** Unique nucleotide mutations occurring exclusively within individual study groups, with frequencies >1%.

**Gene**	**Group A mutations**	**Proportion of total Group A mutations (%)^a^**	**Group B mutations**	**Proportion of total Group B mutations (%)^a^**	**Group C mutations**	**Proportion of total Group C mutations (%)^a^**
ORF1a	20	55.6	27	57.4	65	90.3
ORF1b	11	61.1	15	68.2	32	88.9
S	12	75.0	7	70.0	52	94.5
ORF3a	1	33.3	8	80.0	3	100.0
E	4	80.0	0	0.0	1	100.0
M	1	50.0	2	66.7	3	100.0
ORF6	0	0.0	1	50.0	1	100.0
ORF7a	1	100.0	0	0.0	4	100.0
ORF7b	0	0.0	1	100.0	1	100.0
ORF8	2	100.0	2	100.0	15	93.8
N	6	50.0	8	57.1	16	84.2
ORF10	0	0.0	1	100.0	0	0.0
Full genome	58	60.4	72	63.2	193	91.5

^a^ “Total mutations” refers to all genetic variations present within a group's genome sequences at frequencies exceeding 1%.

### 3.3. Tests for recombination and selection

**Figure 5:**
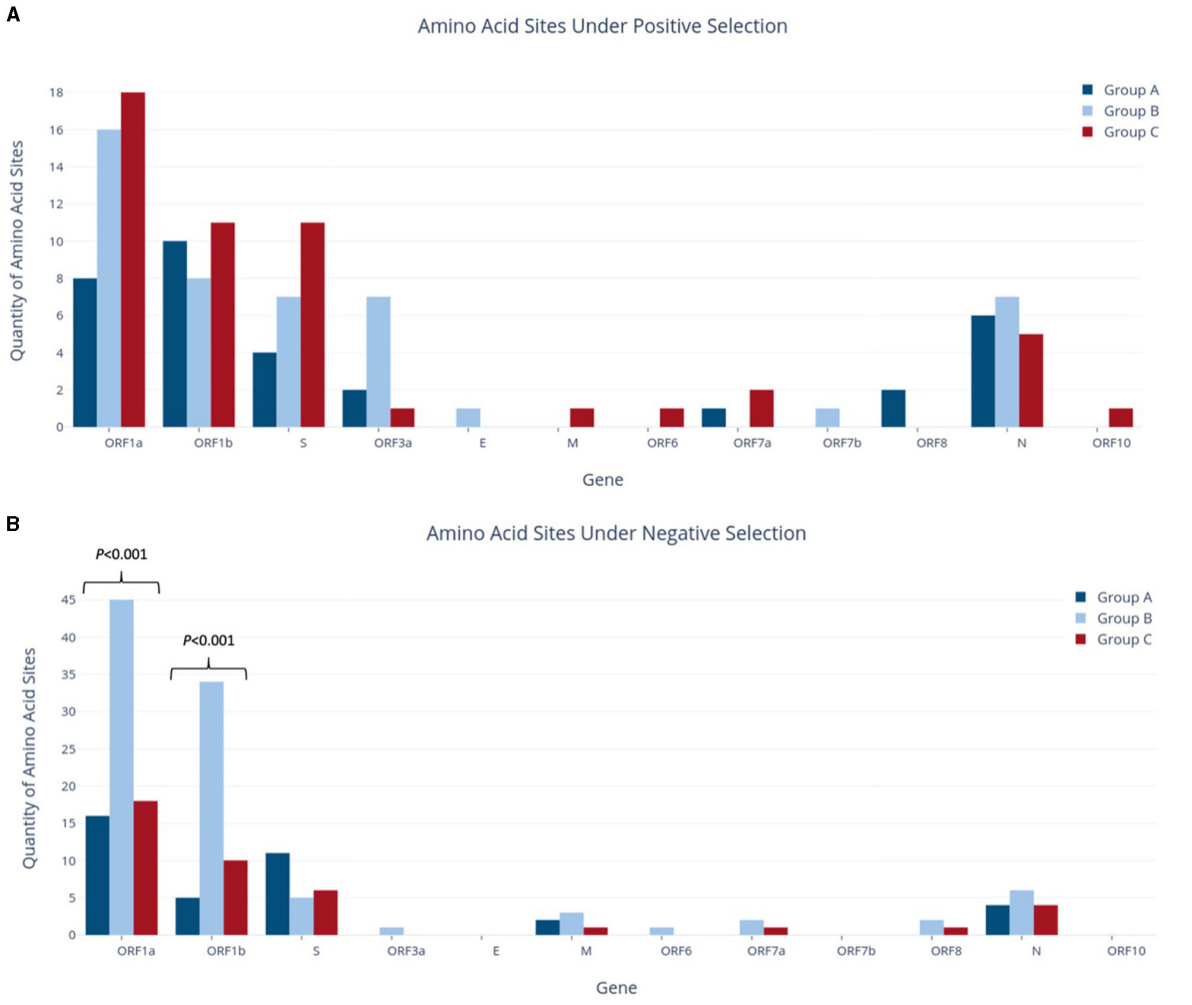
Quantities of amino acid sites identified as experiencing positive selection **(A)** or negative selection **(B)** in study groups.

For each group, individual gene alignments were tested for recombination and then for the presence of positive or negative selection at individual amino acid sites to characterize the evolutionary context of each study group. As Datamonkey tools reject stop codons, the MSA input files were required to have all stop codons replaced with N's. This included the replacement of premature stop codons found in at least one sequence within the ORF1b, S, ORF3a, M, ORF6, ORF7a, ORF7a, ORF7b, ORF8, and N genes in various study groups. Analysis of gene alignments using GARD revealed no evidence of recombination within any individual gene across the study groups, so subsequent testing for positive selection could proceed without further adjustments. The findings from the FUBAR tool, as depicted in Figure 5, indicated that both positive (diversifying) and negative (purifying) selection was detected in all study groups, although the number of amino acid sites reported varied greatly amongst the different genes.

Across the whole genome, the number of amino acid sites experiencing positive selection showed a progressive increase, from 33 sites in Group A to 47 in Group B and 51 in Group C, although no statistically significant difference was observed for any individual genes (Figure 5A). Although Group A had fewer sites under positive selection than Group B, it reported an equivalent number of genes affected. In contrast, Group A had the fewest genes experiencing negative selection, with only five genes vs. the nine and seven genes reported in Groups B and C, respectively. Of note, the ORF1a and S genes in Group A had the highest presence of negative selection, at 16 and 11 sites, respectively. Similarly, ORF1a and ORF1b had the most sites under positive selection, with 8 and 10 sites, respectively. Interestingly, the S gene in Group A had 2.75 times more sites under negative selection than positive selection.

Group B reported a significant increase in the number of sites under negative selection, with 99 sites identified across the whole genome. This represented a substantial difference as compared to Group A, which had 38 sites (2.6 times fewer, *p* < 0.001), and Group C, which had 41 sites (2.4 times fewer, *p* < 0.001). As shown in Figure 5B, most of these sites were found in ORF1a and ORF1b, with 45 and 34 sites, respectively. The difference between study groups was highly significant for both of these genes (*p* < 0.001). As stated above, Group B also had the highest number of genes affected by negative selection out of all groups. ORF1a and ORF1b also contributed the largest number of sites under positive selection in Group B, although on a much smaller scale than those under negative selection (16 and 8 sites, respectively). In contrast to Group A, the S gene in Group B contained more sites under positive selection (seven sites) but fewer sites under negative selection (five sites). Additionally, Group B reported seven sites under positive selection in ORF3a, while Group A reported two sites and Group C reported only one in this gene. This difference in ORF3a was initially statistically significant (*p* < 0.05) but was no longer significant after applying the Bonferroni correction.

Group C had the highest number of amino acid sites under positive selection, with a total of 51 sites across the entire genome. Importantly, Group C was the only group in which the number of sites under positive selection exceeded those under negative selection, as both Groups A and B reported the opposite pattern at the genome level. Group C also found nine genes with sites experiencing positive selection, compared to seven genes in both Groups A and B. ORF1a had an equivalent number of sites under both positive and negative selection (18), while ORF1b was nearly equivalent with 11 sites under positive selection and 10 under negative selection. The S gene in Group C also had a higher number of sites under positive selection (11 sites) as compared to negative selection (six sites), with four of these positive selection sites corresponding to lineage-defining mutations of the Delta VOC (G142D, L452R, P681R, and D950N; Table 3).

**Table 3:** Delta VOC lineage-defining mutations identified in study Groups A–C.

**Gene**	**Mutation**	**Identified groups**	**Selection**	**References**
ORF1a	F924F	A, B, C	Negative (A, B)	^a^
ORF1b	P314L	A, B, C	Positive (A, B, C)	^a^
ORF1b	G662S	A, C	None	^a^
ORF1b	P1000L	C	None	^a^
S	T19R	C	None	^a^, ^b^
S	G142D	C	Positive	^a^
S	E156-	C	None	^a^
S	F157-	C	None	^a^
S	R158G	C	None	^a^
S	L452R	C	Positive	^a^, ^b^
S	T478K	C	None	^a^, ^b^
S	D614G	A, B, C	None	^a^
S	P681R	C	Positive	^a^, ^b^
S	D950N	C	Positive	^a^, ^b^
ORF3a	S26L	C	Positive	^a^, ^b^
M	I82T	C	Positive	^a^, ^b^
ORF7a	V82A	C	None	^a^, ^b^
ORF7a	T120I	C	Positive	^a^, ^b^
ORF8	D119-	C	None	^a^
ORF8	F120-	C	None	^a^
N	D63G	C	Positive	^a^, ^b^
N	R203M	C	None	^a^, ^b^
N	D377Y	C	None	^a^, ^b^

^a^Hodcroft, 2023d at https://covariants.org/variants/21A.Delta.

^b^cov-lineages.org, 2023 at https://cov-lineages.org/global_report_B.1.617.2.html.

To assess the relationship between the sites under positive or negative selection and mutations present at frequencies >1%, comparisons were made within each study group at the amino acid level. The proportion of sites under selection that also had a mutation at the same amino acid site is summarized by the gene in Supplementary Tables 2A–C. As the number of sites under selection varied among genes (Figure 5), numbers were presented as the percentage of sites under selection for ease of comparison. It should be noted that this study focused exclusively on mutations present in >1% of each study group, and consequently, not all sites under selection had corresponding mutations at the same locus that met the >1% threshold. As a result, the overall proportion of selection sites with matching mutations was low across the full genome for all three study groups, although certain genes exhibited higher proportions (Supplementary Tables 2A–C).

Importantly, Group C contained all the expected lineage-defining mutations of the Delta VOC, as described in Table 3 (cov-lineages.org, 2023; Hodcroft, 2023d). Several mutations, including F924F in ORF1a, P314L in ORF1b, and D614G in S, were identified in all study groups, supporting the establishment of these mutations in the viral population at an early stage. In particular, the P314L mutation in ORF1b, which was under positive selection in all three study groups, and the D614G Mutation in S are also lineage-defining mutations for the Alpha, Beta, and Gamma VOCs, all of which were detected earlier than the Delta VOC (Hodcroft, 2023a,b,c,d). The S protein position 681 holds a lineage-defining mutation for both the Alpha and Delta VOCs, although the amino acid substitutions differ between lineages, and was reported to be under positive selection in Group C (Hodcroft, 2023b,d). Similarly, the N protein position 203 serves as a lineage-defining mutation site for the Alpha, Gamma, and Delta VOCs, although the Alpha and Gamma lineages share the R203K substitution while Delta contains R203M (Hodcroft, 2023b,c,d). Additionally, both Group A and C reported the G662S mutation in ORF1b, but this is only a lineage-defining mutation of the Delta and Omicron BA.2.75, XBB, and XBB.1.5 VOCs (equivalent Nextstrain nomenclature are Omicron 22D, 22F, and 23A, respectively), all of which emerged long after the sequence collection dates of Group A (Hodcroft, 2023d,f,g,h).

## 4. Discussion

The error-prone nature of RNA viruses and the massive global availability of susceptible hosts have provided ample opportunity for SARS-CoV-2 to circulate and diversify rapidly (Duarte et al., 2022). Following the succession of the Delta VOC (B.1.617.2) as the dominant global variant, continued adaptive evolution led to the emergence of multiple sub-lineages within the Delta variant family, termed AY lineages by Pango nomenclature (Eales et al., 2022; cov-lineages.org, 2023). Here, we focused on the viral population in India to characterize trends in genetic diversity and selection before and after the emergence of Delta VOC. The range of collection dates for Groups A, B, and C provide distinct snapshots of the viral population at the first introduction of SARS-CoV-2 to India, just before the emergence of the Delta variant, and well after the Delta VOC was established in the country. This unique sampling strategy effectively displays the evolutionary progression toward VOC emergence. To the best of our knowledge, this is currently the only study providing an evolutionary perspective through this tri-phased sampling strategy.

As expected, mutation analyses revealed increasing quantities of mutations with the temporal progression of Groups A–C. Notably, Group C had a significant increase in mutations at both the >1% and >10% thresholds, which is well supported by reports showing that the Delta VOC harbors 29 characteristic mutations that differ from the original Wuhan strain (Khare et al., 2021; Borcard et al., 2022; cov-lineages.org, 2023; Gangavarapu et al., 2023). In particular, 33.2% of the total mutations found in Group C were present in >10% of the group's sequences, as opposed to 16.7% and 15.8% in Groups A and B, respectively. While the increasing quantity of mutations demonstrates an increase in genetic diversity, the larger percentage of mutations reaching the >10% threshold demonstrates the progression toward a new lineage becoming dominant in the viral population. Similarly, intergroup comparisons supported the evolutionary context of Groups A, B, and C. Comparisons of mutations revealed that mutations identified in multiple groups were most often shared between Groups A and B, whereas mutations shared between Groups A and C were rare. This is reasonable given that the collection dates of the groups progressed temporally from Group A to B to C, with Group A and C being collected almost a full year apart. Furthermore, 91.5% of mutations found in >1% of Group C's sequences were unique mutations not shared by any other group, which emphasizes the distinct genetic profile that appears after the emergence of Delta VOC. Despite having lower proportions than Group C, Groups A and B still reported over 60% of their mutations as unique rather than shared, demonstrating that the viral population of each group possesses a specific genetic profile that changes over time.

Another interesting pattern in Group C was the proportion of different mutation types, which differed from Groups A and B. At all examined frequency thresholds (Figure 4), all three groups had a higher percentage of non-synonymous substitutions than synonymous substitutions, but Groups A and B consistently reported similar percentages in each mutation type. While Group C still followed this pattern, the percentages of non-synonymous and synonymous substitutions were distinct, and the proportion of synonymous mutations was lower than those of Groups A and B at all frequencies. This was exchanged for a large increase in deletions, with Group C reporting 12–16 times more deletions than Groups A and B overall (>1% threshold) and additionally being the only group that contained deletions (13 occurrences) above the >10% sequence threshold. Specifically, deletions within the S gene made up 22 of the 48 identified Group C deletions, which is not surprising as S protein deletions have been reported to affect transmissibility, antigenicity, and immune escape, thus conferring a fitness advantage (Harvey et al., 2021; Liu et al., 2021). Deletions have been frequently reported throughout the genome of all five VOC lineages, including the Delta VOC, which dominates Group C (Hodcroft, 2023a,b,c,d,e). No insertions were reported in >1% of sequences in any group, which was unsurprising given that insertions are known to occur far less frequently than deletions in both the SARS-CoV-2 virus and in broader protein evolution (De Jong and Rydén, 1981; Liu et al., 2021). Altogether, Group C has the largest proportion of mutations causing changes in the final amino acid code, such as non-synonymous substitutions and deletions. In total, 74.4% of mutations in Group C at frequencies >1% caused changes in the final amino acid code (vs. 65.6 and 63.2% in Groups A and B, respectively). This proportion increased to 82.9% of mutations at the >10% threshold (vs. 68.8 and 61.1% in Groups A and B, respectively). This tendency to cause a change in the final genome sequence supports the dynamic nature of the SARS-CoV-2 virus and explains its incredible ability to adapt to human populations by continuously diversifying and evolving. Similarly, other RNA viruses such as human immunodeficiency virus 1 (HIV-1) are known to be highly mutable viruses as well due to the activity of the error-prone RNA-dependent RNA polymerase, which creates ample opportunity for diversification (Kustin and Stern, 2021; Duarte et al., 2022).

Certain mutations may become established because of natural selection as favorable changes can provide evolutionary advantages such as improved survival and reproduction of the virus (Karlsson et al., 2014). Shortly after SARS-CoV-2 emergence, selection favored mutations that contributed to reproductive success, such as the S gene D614G substitution, as there was little evolutionary need for antigenic diversity (Carabelli et al., 2023). Interestingly, the D614G mutation was not reported to be under any selection, despite being detected in all study groups (Table 3). This is perhaps because it became an established mutation very early in the pandemic (first reported in January 2020) and was highly prevalent in all our study groups, potentially causing it to be regarded as the standard sequence rather than a mutation (Tian et al., 2021).

As natural and vaccine-acquired immunity against SARS-CoV-2 increased in the host population, so did the need for viral antigenic variations to enable its immune escape and continue transmission (Carabelli et al., 2023). Mutations in viral surface proteins highly exposed to the immune system, such as the S protein, are typically under high selective pressure, and the Delta VOC holds the majority of its characteristic mutations within the S protein (Malik et al., 2021; cov-lineages.org, 2023). For example, the S protein L452R mutation was identified in Group C and found to be under positive selection in our study. Located in the receptor-binding motif region of the RBD, which binds the host angiotensin-converting enzyme-2 (ACE2) receptor, L452R causes structural changes that may stabilize the interaction between the S protein and the ACE2 receptor on the host cell to increase viral infectivity (Tian et al., 2021). Similarly, the S protein P681R mutation was identified in Group C and reported to be experiencing positive selection. This mutation sits within the furin cleavage site of the S protein, and this cleavage of the S1 and S2 subunits is a critical part of host cell entry (Huang et al., 2020; Tian et al., 2021). The P681R mutation in the S protein facilitates furin-mediated cleavage, which improves host cell entry, and was reported to be an important element of succession of Delta VOC over the previously dominant Alpha VOC (Tian et al., 2021; Liu et al., 2022). This list is not exhaustive but conveys the importance of these high-prevalence mutations found in Group C and supports how selection drives the evolution of viral lineages with an advantageous repertoire of mutations. Evidence of both positive and negative selection has been reported by other studies in the S gene, while positive selection has been reported in ORF1ab, ORF3a, and ORF8 (Velazquez-Salinas et al., 2020; Martin et al., 2021; Duarte et al., 2022; Upadhyay et al., 2022).

The number of amino acid sites experiencing positive selection increased with the temporal group progression, from 33 in Group A to 47 in Group B and 51 in Group C. Most of these sites were in ORF1a and ORF1b, which is unsurprising given that they encompass around 70% of the viral genome together (Bai et al., 2022). Other genes reporting high numbers of variations in all groups included S and N, while the remaining structural genes (E and M) found hardly any sites under positive selection. Interestingly, Group B was the only group to report that ORF3a (functions in viral release, inflammasome activation, and necrotic cell death) had a higher number of sites under positive selection, equivalent to the S and N genes (Naqvi et al., 2020; Gorkhali et al., 2021; Bai et al., 2022). In terms of negative selection, Group B had 2.4–2.6 times more sites detected within the whole genome than any other group, with ORF1a and ORF1b contributing a large number of sites. ORF1b notably reported a jump from 5 sites in Group A to 10 sites in Group C and 34 sites in Group B. Additionally, Group B contains more mutations reaching the >1% threshold than Group A, but fewer mutations reaching the >10% threshold. Given that Group B represents an intermediate period where SARS-CoV-2 is established and circulating in the country, but the Delta VOC has not yet emerged, we suspect that these results demonstrate a period of heightened diversification within the viral population.

As the virus moves away from its initial introduction in India (Group A), it gains more opportunities to diversify by increasing the number of infected hosts and by the progression of time, allowing more viral replication and transmission cycles (Carabelli et al., 2023). This heightened genetic diversity is evident in Group B as characterized by an increased total mutation count; however, many of these did not reach the >10% threshold, which indicates the presence of numerous low-prevalence lineages rather than a single dominant lineage. Similarly, Group B reported the highest proportion of synonymous mutations, which do not change the translated amino acid code. This observation aligns with the findings that Group B had the largest number of sites experiencing negative selection across all study groups. During viral evolution, negative selection plays a crucial role in preserving essential functional features through synonymous substitutions (Spielman et al., 2019; Berrio et al., 2020). As RNA viruses are known to experience strong purifying selection due to their densely coded genome, it is plausible that the observed increase in Group B's negative selection is working to maintain essential gene function while the viral population is undergoing a massive expansion and diversifying into new lineages (Kustin and Stern, 2021). This notion is supported by the work of Martin et al., who reported a significant shift in selective pressures within the global SARS-CoV-2 viral population about 11 months after its initial appearance (November 2020), where the number of sites detected under both positive and negative selection increased substantially and continued throughout the following months in 2021. Remarkably, the time frame in the study by Martin et al. coincides with the collection dates of Groups B and C in our work, further supporting the differentiation of Groups B and C from Group A with respect to the quantities of sites under selection.

One notable limitation of this study is its dependency on the GISAID database for viral genome availability and quality. The detection of viral variants heavily depends on the extent of local sequencing efforts, the throughput capacity of Indian sequencing laboratories, and the consistent deposition of Indian viral sequences in the GISAID database. We encountered challenges during the study of Group A, which was sampled during the early stage of the COVID-19 pandemic in India when viral genome availability in GISAID was very limited. Consequently, both Groups A and B were sampled over much wider collection date ranges than Group C to provide a similar number of genomes across all groups. This suggests a less precise snapshot of genetic diversity as the wider ranges of collection dates may include multiple cycles of viral transmission. Similarly, our study relied on the availability of high-quality sequences within databases. Despite applying quality filters during database queries, many samples were removed from our study due to the presence of unreliable genome regions suspected of poor-quality sequencing and/or amplicon dropout. This was especially troublesome in Group C, where the increased prevalence of mutations might have made sequencing with previous primer schemes increasingly difficult, which was a known problem for the Delta VOC (Borcard et al., 2022). According to the periods of sequence collection, Group A may have used ARTIC primer V2 or earlier, while Group B and C may have used ARTIC primer V3 or earlier (Quick, 2020). The ARTIC primer V4 series, designed to address SARS-CoV-2 Beta, Gamma, and Delta VOC mutations in V3 primer binding sites, was released on June 18, 2021, which was several months after the collection dates of Group C (Davis et al., 2021). Nonetheless, we endeavored to address these limitations through the utilization of a larger number of genomes within each group, meticulous attention to genome quality, and a focus on whole-genome analysis.

This study effectively demonstrates the evolutionary progression toward VOC emergence in SARS-CoV-2, with a specific focus on the Delta VOC, known for its significant global impact as one of the most virulent variant lineages. With a unique tri-phased sampling strategy, this study provides valuable insight into the evolutionary dynamics preceding and following the emergence of Delta VOC. It is plausible that similar patterns of genetic diversity and evolutionary selection may have been observed in other VOC lineages before their initial appearance. By enhancing our understanding of the dynamic nature of SARS-CoV-2 evolution, this study has the potential to facilitate earlier recognition and prediction of the emergence of future variant lineages. Such insights could be instrumental in mitigating the impact of the emerging variants and effectively responding to the evolving challenges from the SARS-CoV-2 VOCs.

## Data availability statement

The original contributions presented in the study are included in the article/Supplementary material, further inquiries can be directed to the corresponding author.

## Author contributions

KL: conceptualization and formal analysis. KL and SM: methodology and writing—original draft preparation. KL, SM, PS, and HJ: writing—review and editing. All authors have read and agreed to the published version of the manuscript.
