# Supplementary material for: Tracking the evolution of the SARS-CoV-2 Delta variant of concern: analysis of genetic diversity and selection across the whole viral genome
Source: Front Microbiol. 2023 Aug 8;14:1222301. doi: 10.3389/fmicb.2023.1222301 (PMC10443222; doi:10.3389/fmicb.2023.1222301)
Supplement: Supplementary file 1 [file Table_1.DOCX]

Supplementary Material

Tracking the Evolution of the SARS-CoV-2 Delta Variant of Concern: Analysis of Genetic Diversity and Selection Across the Whole Viral Genome

Katherine Li, Stephanie Melnychuk, Paul Sandstrom, Hezhao Ji*

*** Correspondence:** Hezhao Ji: hezhao.ji@phac-aspc.gc.ca

# Supplementary Data

**Supplementary Table 1A.** Genetic diversity detailed summary of Group A by frequency and type of codon mutation type per gene.

|  | ORF1a | ORF1b | S | ORF3a | E | M | ORF6 | ORF7a | ORF7b | ORF8 | N | ORF10 |
| --- | --- | --- | --- | --- | --- | --- | --- | --- | --- | --- | --- | --- |
| 1-10% |  |  |  |  |  |  |  |  |  |  |  |  |
| Total Mutations | 31 | 15 | 14 | 2 | 5 | 1 | 1 | 1 | 0 | 2 | 8 | 0 |
| Non-synonymous | 17 | 9 | 9 | 2 | 1 | 1 | 0 | 1 | 0 | 2 | 6 | 0 |
| Synonymous | 14 | 6 | 5 | 0 | 0 | 0 | 1 | 0 | 0 | 0 | 2 | 0 |
| Insertions | 0 | 0 | 0 | 0 | 0 | 0 | 0 | 0 | 0 | 0 | 0 | 0 |
| Deletions | 0 | 0 | 0 | 0 | 4 | 0 | 0 | 0 | 0 | 0 | 0 | 0 |
| >10% |  |  |  |  |  |  |  |  |  |  |  |  |
| Total Mutations | 5 | 3 | 2 | 1 | 0 | 1 | 0 | 0 | 0 | 0 | 4 | 0 |
| Non-synonymous | 3 | 2 | 1 | 1 | 0 | 0 | 0 | 0 | 0 | 0 | 4 | 0 |
| Synonymous | 2 | 1 | 1 | 0 | 0 | 1 | 0 | 0 | 0 | 0 | 0 | 0 |
| Insertions | 0 | 0 | 0 | 0 | 0 | 0 | 0 | 0 | 0 | 0 | 0 | 0 |
| Deletions | 0 | 0 | 0 | 0 | 0 | 0 | 0 | 0 | 0 | 0 | 0 | 0 |

**Supplementary Table 1B.** Genetic diversity detailed summary of Group B by frequency and type of codon mutation type per gene.

|  | ORF1a | ORF1b | S | ORF3a | E | M | ORF6 | ORF7a | ORF7b | ORF8 | N | ORF10 |
| --- | --- | --- | --- | --- | --- | --- | --- | --- | --- | --- | --- | --- |
| 1-10% |  |  |  |  |  |  |  |  |  |  |  |  |
| Total Mutations | 41 | 19 | 8 | 8 | 1 | 2 | 2 | 0 | 1 | 3 | 10 | 1 |
| Non-synonymous | 21 | 13 | 5 | 8 | 1 | 0 | 0 | 0 | 1 | 3 | 5 | 1 |
| Synonymous | 20 | 6 | 2 | 0 | 0 | 2 | 2 | 0 | 0 | 0 | 3 | 0 |
| Insertions | 0 | 0 | 0 | 0 | 0 | 0 | 0 | 0 | 0 | 0 | 0 | 0 |
| Deletions | 0 | 0 | 1 | 0 | 0 | 0 | 0 | 0 | 0 | 0 | 2 | 0 |
| >10% |  |  |  |  |  |  |  |  |  |  |  |  |
| Total Mutations | 6 | 3 | 2 | 2 | 0 | 1 | 0 | 0 | 0 | 0 | 4 | 0 |
| Non-synonymous | 3 | 1 | 1 | 2 | 0 | 0 | 0 | 0 | 0 | 0 | 4 | 0 |
| Synonymous | 3 | 2 | 1 | 0 | 0 | 1 | 0 | 0 | 0 | 0 | 0 | 0 |
| Insertions | 0 | 0 | 0 | 0 | 0 | 0 | 0 | 0 | 0 | 0 | 0 | 0 |
| Deletions | 0 | 0 | 0 | 0 | 0 | 0 | 0 | 0 | 0 | 0 | 0 | 0 |

**Supplementary Table 1C.** Genetic diversity detailed summary of Group C by frequency and type of codon mutation type per gene.

|  | ORF1a | ORF1b | S | ORF3a | E | M | ORF6 | ORF7a | ORF7b | ORF8 | N | ORF10 |
| --- | --- | --- | --- | --- | --- | --- | --- | --- | --- | --- | --- | --- |
| 1-10% |  |  |  |  |  |  |  |  |  |  |  |  |
| Total Mutations | 52 | 26 | 34 | 2 | 1 | 0 | 0 | 1 | 0 | 10 | 15 | 0 |
| Non-synonymous | 13 | 11 | 16 | 2 | 1 | 0 | 0 | 1 | 0 | 7 | 13 | 0 |
| Synonymous | 21 | 14 | 3 | 0 | 0 | 0 | 0 | 0 | 0 | 2 | 2 | 0 |
| Insertions | 0 | 0 | 0 | 0 | 0 | 0 | 0 | 0 | 0 | 0 | 0 | 0 |
| Deletions | 18 | 1 | 15 | 0 | 0 | 0 | 0 | 0 | 0 | 1 | 0 | 0 |
| >10% |  |  |  |  |  |  |  |  |  |  |  |  |
| Total Mutations | 20 | 10 | 21 | 1 | 0 | 3 | 1 | 3 | 1 | 6 | 4 | 0 |
| Non-synonymous | 12 | 9 | 12 | 1 | 0 | 2 | 1 | 3 | 1 | 0 | 4 | 0 |
| Synonymous | 8 | 1 | 2 | 0 | 0 | 1 | 0 | 0 | 0 | 0 | 0 | 0 |
| Insertions | 0 | 0 | 0 | 0 | 0 | 0 | 0 | 0 | 0 | 0 | 0 | 0 |
| Deletions | 0 | 0 | 7 | 0 | 0 | 0 | 0 | 0 | 0 | 6 | 0 | 0 |

**Supplementary Table 2A.** Group A: Percentage of amino acid sites under selection that are associated with a mutation in >1% of sequences at the same locus.

|  | Positively selected sites with a corresponding mutation (%) | Negatively selected sites with a corresponding mutation (%) | Total sites (positive/negative) under selection with a corresponding mutation (%) |
| --- | --- | --- | --- |
| ORF1a | 37.5 | 56.3 | 50.0 |
| ORF1b | 40.0 | 80.0 | 53.3 |
| S | 25.0 | 45.5 | 33.3 |
| ORF3a | 50.0 | n/a ^a^ | 50.0 |
| E | n/a ^a^ | n/a ^a^ | n/a ^a^ |
| M | n/a ^a^ | 0.0 | 0.0 |
| ORF6 | n/a ^a^ | n/a ^a^ | n/a ^a^ |
| ORF7a | 0.0 | n/a ^a^ | 0.0 |
| ORF7b | n/a ^a^ | n/a ^a^ | n/a ^a^ |
| ORF8 | 0.0 | n/a ^a^ | 0.0 |
| N | 50.0 | 25.0 | 40.0 |
| ORF10 | n/a ^a^ | n/a ^a^ | n/a ^a^ |
| Full Genome | 35.3 | 50.0 | 41.7 |

^a^ n/a indicates no selection events recorded for the specified gene.

**Supplementary Table 2B.** Group B: Percentage of amino acid sites under selection that are associated with a mutation in >1% of sequences at the same locus.

|  | Positively selected sites with a corresponding mutation (%) | Negatively selected sites with a corresponding mutation (%) | Total sites (positive/negative) under selection with a corresponding mutation (%) |
| --- | --- | --- | --- |
| ORF1a | 50.0 | 22.2 | 29.5 |
| ORF1b | 62.5 | 14.7 | 23.8 |
| S | 28.6 | 40.0 | 33.3 |
| ORF3a | 42.9 | 100.0 | 50.0 |
| E | 0.0 | n/a ^a^ | 0.0 |
| M | n/a ^a^ | 33.3 | 33.3 |
| ORF6 | n/a ^a^ | 0.0 | 0.0 |
| ORF7a | n/a ^a^ | 0.0 | 0.0 |
| ORF7b | 0.0 | n/a ^a^ | 0.0 |
| ORF8 | n/a ^a^ | 0.0 | 0.0 |
| N | 14.3 | 0.0 | 7.7 |
| ORF10 | n/a ^a^ | n/a ^a^ | n/a ^a^ |
| Full Genome | 38.0 | 18.2 | 24.8 |

^a^ n/a indicates no selection events recorded for the specified gene.

**Supplementary Table 2C.** Group C: Percentage of amino acid sites under selection that are associated with a mutation in >1% of sequences at the same locus.

|  | Positively selected sites with a corresponding mutation (%) | Negatively selected sites with a corresponding mutation (%) | Total sites (positive/negative) under selection with a corresponding mutation (%) |
| --- | --- | --- | --- |
| ORF1a | 38.9 | 50.0 | 44.4 |
| ORF1b | 36.4 | 20.0 | 28.6 |
| S | 90.9 | 50.0 | 76.5 |
| ORF3a | 100.0 | n/a ^a^ | 100.0 |
| E | n/a ^a^ | n/a ^a^ | n/a ^a^ |
| M | 100.0 | 100.0 | 100.0 |
| ORF6 | n/a ^a^ | 0.0 | 0.0 |
| ORF7a | 50.0 | 0.0 | 33.3 |
| ORF7b | n/a ^a^ | n/a ^a^ | n/a ^a^ |
| ORF8 | n/a ^a^ | 0.0 | 0.0 |
| N | 80.0 | 0.0 | 44.4 |
| ORF10 | 0.0 | n/a ^a^ | 0.0 |
| Full Genome | 56.0 | 35.7 | 46.7 |

^a^ n/a indicates no selection events recorded for the specified gene.
